# “A magical little pill that will relieve you of your womanly issues”: What young women say about menstrual suppression

**DOI:** 10.3402/qhw.v11.32932

**Published:** 2016-11-23

**Authors:** Colleen McMillan, Amanda Jenkins

**Affiliations:** 1 School of Social Work, Renison University College, University of Waterloo, Waterloo, ON, Canada; 2 Department of Applied Psychology, University of Guelph, Guelph, ON, Canada

**Keywords:** Menstruation, healthy, menstrual suppression, marketing, discourse analysis, feminism, agency, gender

## Abstract

Perceptions of menstruation by media discourses portray this bodily function to be messy, inconvenient, and as an unnecessary phenomenon to be controlled or possibly eliminated. Commercials shown on YouTube targeted toward young women suggest that having a monthly period is not healthy and a lifestyle that is menses free is both pharmacologically available and recommended in order to live a fuller life. We explored the meanings attached to online menstrual suppression commercials with 10 women aged between 18 and 25. In-depth open-ended interviews were conducted over a 10-month period in 2014 after each participant viewed three menstrual suppression online advertisements. Feminist critical discourse was used for analysis with both authors coding for inter-rater reliability recognizing how our age difference and relationship as mother and daughter informed our interpretation. An overarching theme of tension emerged from the interviews with participants feeling detached due to the gendered stereotypes the commercials used to frame menstruation as compared to their own lived experience. Meanings associated with the menstrual suppression commercials were contrary to the participants’ lived experience of menstruation as a healthy process not a detrimental one to their well-being as suggested by the commercials. Subliminal messages within the advertisements were identified as reinforcing gender bias and prejudices, including those associated with femininity. Despite attempting to emulate popular culture, the menstrual suppression advertisements were largely dismissed by this group of participants as undermining their intelligence and of intentionally creating divisive binaries between groups of women. This study suggests that historical bias and stereotypical prejudices were identified by this group of young women within the marketing of menstrual suppression products and, as such, were dismissed as inauthentic to the menstruation experience reflecting a form of menstrual activism.

Menstruation is a process that women experience throughout most of their lives and is an embodied symbol of womanhood, fertility, and health. Yet, menstruation in Western society is constructed as bothersome, inconvenient, messy, shameful, and even unhealthy. Historically, Western medical literature considered menstruation a physiological deficit with physicians viewing menstruation as pathological and incapacitating for women (Zieff, 2010) since ancient Greece (Parker, 1983). Described as “a needless loss of blood” (Johnston-Robledo, Ball, Lautra, & Zekoll, 2003, p. 60) and “harmful” (Coutinho, 1999, p. 195), menstruation was spoke of a sick time (Zieff, 2010) during the late 19th and early 20th century in the United States with described physical incapacities so severe that women were advised to stay confined to the home and preferably in bed. The deficit perception of menstruation as harmful moved into the mental disorders realm as evidenced by the inclusion of premenstrual syndrome symptoms (PMS) and premenstrual dysphoric disorder (PMDD) in the 5th edition of the Diagnostic and Statistical Manual of Mental Disorders (DSM) published in 2013. Diagnostic criteria including “marked anger, interpersonal conflicts, difficulty in concentration, insomnia and a subjective sense of being overwhelmed or out of control” (American Psychiatric Association, 2013) have been used as a legal defence dating back to 1843 when an English court deemed that menstruating women could indeed be considered insane during the last week of the luteal phase. Earlier studies held menstruating women accountable for inciting civil disobedience, failing in school, running away from home, and increasing crime (Fausto-Sterling, 1985). The intersection of medicine and law has been a critical one due to the essential role played by physicians in defining the legal significance of menstruation within the criminal justice system since the early 1980s. Within political realms, menstruation and PMS continue to be used by political leaders as a way to discredit women. As a recent example, a female reporter who challenged the current US republican candidate regarding his lack of debating skills was publically dismissed with a statement inferring her lack of intelligence was due to her menstruating state.

Aside from the medical, legal and political ramifications linked to menstruation, social and cultural stigma has long been attached to the menstruating body. Feminists including Bobel (2006), Chrisler (2011), Grosz (1994), and Ussher (2006), among others, note historically that menstruation was viewed “with shame, embarrassment, disgust, [holding] the powers of contamination” (Grosz, 1994, p. 206). Constructed as something uncontrollable, uncontainable, and capable of producing feelings of loathing and fear (Bouson, 2009), the menstruating female body inhabits a tenuous space between being concurrently revered or repulsed. Such messages shame women into concealing signs of the menstruating body and to become hypervigilant about self-surveillance. A study by Kowalski and Chapple (2000) explored how the internalization of shaming norms was understood by women who were menstruating. The majority of the women felt a male interviewer would view them less favorably if he knew they were menstruating. This finding speaks to the perceived necessity of concealment that goes beyond visual olfactory signs of menstruation such as spotting or odor. It also speaks to insinuated insecurities around menstruation as a normal and physiologically healthy process, or as Mamo and Fosket (2009) wrote “a process that is fundamentally at odds with women's ability to function normally” (p. 930).

As part of what Mamo and Fosket (2009) label as “an ongoing biomedicalization that emphasizes risk reduction and management” (p. 925), the emergence of a new class of pharmaceuticals arrived on the market in 2003 aimed at reducing the menstrual cycle to four periods per year. Referred to as extended-cycle menstrual suppression, commercials were constructed around messages of convenience and lifestyle taking aim at younger women (Eldridge, 2010). While direct-to-consumer advertising of drugs through television is not legal in Canada, as compared to the Unites States, information and marketing of oral contraceptives were widely available and directed toward young women through the Internet. Within Canada, over 44% of women of reproductive age use oral contraceptives as a means of birth control according to the Canadian Contraception Consensus (Black et al., 2015). This percentage, however, may be underestimated, as women who take oral contraceptives for reasons beyond preventing pregnancy, such as menstrual regulation or acne, are excluded from this report (Jones, 2011).

Branded as lifestyle drugs (Watkins, 2012), products such as Yaz by Bayer Healthcare Pharmaceuticals, and Seasonale and Seasonique, the second-generation drug, by Duramed Pharmaceutical, were advertised as “new pharmacological therapies” designed to improve the quality of one's life (Mamo & Fosket, 2009, p. 925) and to boost personal productivity. Such advertisements shifted the message away from oral contraceptives as a birth control method to one of a lifestyle choice by taking a pill to eliminate the inconvenience and unpleasantness of menstruation, such as headaches, moodiness, acne, and irritability (Singer, 2009). The refashioning of the female body as non-menstruating was presented as the new ideal readily available through a prescription (Johnston, Barnack, & Wares, 2006; Fahs, 2013; Johnston-Robledo, Barnack, & Wares, 2006; Rose, Joan, & Couture, 2008). Emphasizing the lifestyle aspect of medical suppression while exonerating documented known and yet unknown health risks placed coming of age generations of young women at physical jeopardy. Physician and author Dr. Susan Rako writes that menstrual suppression is particularly harmful to “the thinning of adolescents’ and young women's bones” in particular, placing this group at a higher risk for osteoporosis (Rako, 2006, p. 20). Packaged as a solution to menstrual-related stigma, menstrual suppression products are thought to be particularly “attractive to women who have not yet developed comfort with menstrual management and are socialised to see their flow as an unnecessary nuisance” (Society for Menstrual Cycle Research, 2011, p. 3). Furthermore, menarchists charge that pharmaceutical techniques framing menstrual suppression as a “feminist dream” is a fallacy; insisting that true freedom comes in societal messages of acceptance versus stigma and shaming (Docherty, 2010).

To better understand how young women view and uptake these messages from menstrual suppression advertisements today, we turned to a feminist critical discourse lens to identify, name, and unpack the embedded discourses around online menstrual suppression commercials. The goal of this study was to understand the meanings young women attach to menstrual suppression and how these messages might inform their decision-making regarding their use. We define menstrual suppression in our article as the use of oral contraceptives to prevent menstruation from occurring either in its normal frequency or interrupting the process for extended periods of time (Society for Menstrual Cycle Research, 2011).

As academics in Psychology and Social Work and clinical practitioners, we approach this topic making our individual standpoints transparent to reflect our feminist values and paradigm of practice. We are a mother and daughter and menstruating women. The differences in our age bookmark two diverse experiences of using oral contraceptives: 35 years ago as a birth control method for the mother and currently as a lifestyle option for the daughter.

## Methodology

Qualitative methodology seeks a deeper understanding of a phenomenon and is guided by the philosophical assumption that knowledge lies within the meanings that people make of the phenomenon. We framed our study from a feminist constructivist approach in order to reach a deeper subjective understanding of how our participants made sense of the online commercials promoting menstrual suppression. Constructivists pose questions that are more nuanced and include a reflexive stance by the researcher making the method suited to the exploration of sensitive topics (McMillan, 2010). Feminist critical discourse analysis (FCDA) was used to remain congruent to our epistemological framework of inquiry (Lazar, 2007). As an analytic approach, FCDA is concerned around the way in which social power, dominance, and structural inequality including that of gender are reproduced and resisted through social discourses (Van Dijk, 2014). The question of how many participants are necessary for a qualitative study was addressed in two ways; a review of the literature (Baker & Edwards, 2012; Crouch & McKenzie, 2006) and that of reaching saturation (Fusch & Ness, 2015).

Ethics approval was received from the (University of Waterloo) and all participants provided written consent for the audio taping of the interviews. The use of a tape recorder was disclosed, both for ethical as well as methodological reasons, as it may impact the conversation and alter the context of what is said (Warren & Karner, 2000).

### Sample

Both purposive and convenience sampling were done to recruit 10 women within the age group of 18–25. This age group was intentionally chosen to capture how culturally constructed messages around menstruation are experienced and interpreted. In addition, decision-making around reproductive and menstruation choices gain greater developmental importance during these ages, characterized by issues of independence, dating, and sexuality (Newton & Hoggart, 2015; Smetana, 1981). Posters were placed in the health services waiting room at a university campus where the authors were situated. Participants were also asked if they had friends who might be interested participating in the study. As a goal of qualitative research is to gain a deeper understanding of the meaning of a phenomenon, purposive sampling is supported (Creswell, 1998). Of our 10 participants, 4 were Anglo-Saxon, 3 were Chinese, 1 was Iranian, 1 was Irish, and 1 was Korean. The median age was 21.8 years. Six participants were enrolled in the humanities, three in health sciences, and one in a business program. The participants did not receive any remuneration so that their decision to be interviewed was completely voluntary.

### Procedure

Drawing upon the literature (Agee, 2009; Charmaz, 2006; Flick, 2006), we intentionally opened up our interviews with broad general questions starting with menstruation before transitioning into menstrual suppression. We were guided by the writings of Maxwell (2005) who cautioned starting with questions that are “too focused” that can inhibit a researcher's understanding and analysis. We also felt that using our first three “discovery-oriented” questions (Maxwell, 2005, p. 67) would help guide our process for creating more nuanced and refined probes while remaining reflexive. After the participants were asked what they knew about the term menstrual suppression, three online commercials featuring oral contraceptives aimed to interrupt menstrual flow were showcased. Criteria used to select the commercials included the following inclusions: mentioning of PMS, PMDD, treatment of these symptoms, and the use of negative descriptors in reference to menstruation. Content in the three chosen commercials followed a similar format. This involved showcasing of women experiencing physical menstrual symptoms, a screenshot of the menstrual suppression product followed by the same actors devoid of symptoms, and ending with advice being given from medical practitioners. The stated side effects associated with the oral contraceptives in these commercials were consistent in wording that stated, “heart attack and/or stroke, deep vein thrombosis, cerebrovascular accidents, pulmonary embolism, kidney failure or problems, gallbladder removal, severe migraines among others” and were verbal with no pictorial accompaniment in comparison to the depiction of the menstrual symptoms. As mentioned, the commercials included Yaz, Seasonale and Seasonique (approved by Food and Drug Administration (FDA) in 2006). After watching the three commercials, each participant was asked the questions found in Table I.

**Table I:** Questions asked of participants.

1. What are the words that you associate with menstruation?
2. What is your understanding of the words “menstrual suppression?”
3. What is your understanding based upon?
4. Do you think it is healthy/unhealthy for menstruation to be suppressed? Why or why not?
5. Do you think it is healthy/unhealthy for a woman to have four periods per year?
6. Do these commercials on birth control change your perspective on menstruation?
7. Is there anything else you would like to comment on about the commercials?

Each author augmented the audio-recording with hand-written field notes following each interview, noting possible content that could be used as probes to extend questions toward promoting a deeper understanding (Maxwell, 2005; Wolfinger, 2002). As an extra step toward reflexivity, we contrasted and compared our field notes in how such probes were used (Jacob & Furgersin, 2012). Since FCDA framed our study, each researcher was sensitive to the terms chosen by the participants, listening for words that could then be incorporated into a probe. Alternatively, we practiced attentiveness to spaces of silence, as indications of ambivalence or uncertainty that might hold unspoken content (McMillan, 2010).

### Data analysis

We were drawn to FCDA as a framework in which to work with our data for its emphasis on “critiquing discourses which sustain a patriarchal social order. FCDA is a political perspective on gender, concerned with demystifying the inter-relationships of gender, power, and ideology in discourse” (Lazar, 2005, p. 5).

In-depth, semi-structured interviews between 1 and 3 h were facilitated, digitally recorded, and transcribed by both authors who followed Charmaz's (2005) approach of line-by-line coding in order to stay close to the data, be sensitive to emerging themes, and remain congruent to a feminist critical discourse framework as outlined by Lazar (2005). Each interview generated between 16 and 22 pages of single-spaced transcription, ensuring that the data collected were rich and allowed for a deeper understanding of the meanings (Gill, Stewart, Treasure, & Chadwick, 2008) associated with our topic of menstrual suppression. Our goal was to capture emerging nuances embedded in the transcriptions regarding understandings and meanings associated with menstrual suppression by our participants. We were also interested in knowing how participants negotiated understanding menstruation as being healthy or normal or that of being unhealthy and abnormal as suggested by the three menstrual suppression commercials.

After line-by-line coding, the two researchers independently engaged the concept of theoretical sensitivity, a process typically associated with grounded theory. We felt the concept of theoretical sensitivity was uniquely relevant to our study, particularly the theoretical categories pertaining to literature and personal experience (Corbin & Strauss, 2014). Comparing and contrasting the initial word clusters identified through the line-by-line coding with content found in the literature contextualized emerging meanings. Strauss and Corbin (1990) advocate for this additional stage as a way of carefully looking at the data as a way to explore all of the dimensions of a phenomenon that may escape a researcher (pp. 75–79) therefore adding rigor to the coding stage.

What followed next was several iterations of collapsing codes into categories. We were mindful during the coding of how similar the meanings derived from the transcriptions were, and so to address the possibility of confirmation bias we experimented with different hierarchical coding (also known as tree coding) to ensure that the meanings were solidly grounded in the data. This was a very useful exercise as it supported the surfacing of meanings that were dissimilar and created space for discrepancies to be seen, significant for alternative interpretations of meanings to be considered.

When a discrepancy was discovered, the authors jointly returned to the transcriptions to inquire further into possible meanings reflective of FCDA, in addition to revisiting our field notes. We were also intentional of how our different standpoints informed our interpretation, and hence were diligent in practicing reflexivity as defined by Alvesson and Skoldberg (2009). This constant cross-checking reflected our attempt to be mindful of bias and reflective of our multiple positions of academics, practitioners, women, and mother and daughter. Each researcher coded separately for purposes of inter-rate reliability, mindful of how our individual standpoints of menstruating women, positioned at opposite ends of the menstrual lifecycle, informed our coding which will be commented on in the discussion section.

## Results

The purpose of this study was to explore young adult women's understanding of menstrual suppression through the viewing of menstrual suppression advertisements. The overarching questions we explored in this article were the following: What are the meanings attached to menstrual suppression by young women? How do these meanings inform their decision-making regarding menstrual suppression products?

Meanings that emerged from the transcriptions extended beyond what we had expected, in that responses were rich and complex, speaking to the deeper issues encased within the marketing commercials. The iterative coding process began with 467 subthemes that were eventually collapsed into four main themes from the data; portraits of knowledge holders, the personification of menstruation, inferred failings of women (including that of weight), and the fear versus freedom. To show coding transparency between the questions asked of the participants and the collapsing of codes into the final four themes, *in vivo* quotes will alternative with text. Lastly, interpretations by the two researchers were member checked to ensure construct validity as defined by Koelsch (2013).

### Theme 1—What a knowledge holder looks like

All 10 of the participants commented on how role casting within the commercials constructed specific yet different “knowledge holders” resulting in information some women may use in their decision-making process on whether to use oral menstrual suppression drugs. It was noticed that a white female physician described the medical benefits of menstrual suppression using clinical and detached language, which infers a privileged type of knowledge. After watching the Seasonique commercial, one participant [P4] worded it this way, “they use a medical doctor who actually said ‘there is no medical need to have a monthly period,’” also noticing the physician used a more definitive tone when speaking. All 10 participants agreed that consumers would link a higher threshold of creditability associated with a physician spokesperson.

Conversely, in the same commercial, a layperson spoke to the list of side effects associated with menstrual suppression drugs. The decision by the marketers to use a layperson to speak to the side effects was felt by the participants to be deliberate in order to minimalize the severity of such outcomes. This last comment is timely as it relates to a recent case–control study of 15,545 women whose risk factor for venous thromboembolism was increased four times when exposed to birth control ingredients drospirenone, gestodene, cyproterone, and desogestrel (Vinogradova, Coupland, & Hippisley-Cox, 2015).

In addition as to who was cast in the roles of physician and consumer, eight of the participants also noted contextual issues such as voice tone, pace of speaking, and physical appearance. The speed in which content was delivered was noted by a participant [P2] as polarized between the physician and the layperson as supported by her statement,

they [the physicians] always seem to speed up a lot faster when talking about all the consequences and these consequences are actually what we as women want to know. We do want to know about the benefits of taking birth control pills but what we really need to be concerned about is how it is going to affect our bodies.

Similarly, it was noted that a deeper voice was attached to the “expert” or privileged knowledge holder as illustrated by the following quote by another participant [P9]:

for the female doctor her voice is a lot deeper than the girl who has blonde hair [the consumer]. Not all blondes are bubbly and high pitched and not all brunettes or dark haired are more reasonable people. That's a really unfair portrayal and then in conclusion so what is the actual purpose of these pills?

Each of the participants noted the intersectionality of race, visibility, hair color, and intelligence within the three commercials. It was noted that while the commercials made a passive nod toward diversity, the attempts drew the viewer's attention to the lack of, rather than an attempt for inclusivity, as noted by a sentence from participant [P5] “there is always one black woman and they are never the centre.”

Lastly, the linking of stereotypical physical characteristics to that of intellect was pointed out by seven of the participants as being offensive, as seen by the following quote by one of the participants [P1]:

they start out by using someone who has blonde hair and in the media. They typically portray someone who is fair skinned and blonde haired women as less intelligent … they also show her as the emotional wreck. The more sensible one in the commercial is the brunette with the medical degree.

This finding is consistent with a study by Kyle and Mahler (2010) where college students associated women with brunette hair as being more capable and successful in their careers. In the three commercials shown to the participants, two of the expert knowledge holders were physicians and one was a lawyer, all three having brunette hair.

### Theme 2—Personification of menstruation—“this is healthy?”

In the Seasonale commercial, menstruation is personified by an older woman dressed in a matronly green, shiny business suit, wearing pearl earrings with coiffed hair. She is referred to as “Mother Nature” and is seen either lurking or stalking young women on a beach, at a party or in one case ready to embark on a honeymoon. The contrasting of age between the younger women and Mother Nature was noted by nine of the participants as a way to construct menstruation, or as one participant [P2] said, “a really ugly woman.” One of the participants [P4] did not find the actress either attractive or unattractive—but rather, “out of date with how she is made to look.” The depiction of what was constructed as natural, “it says something about how natural or healthy looks like,” and of a likely outcome “they have mother flow depicted as this really horrible, ugly woman who's coming to wreck your life” was identified as being incongruent by one of the participants [P7]. In the commercial, younger women are seeking avenues to hide or places to escape the clutches of Mother Nature who has an uncanny ability to appear from nowhere from behind large shrubs. Nine of the participants noted the antiquated look of the woman cast as Mother Nature demonstrated through clothing and hair that resembled a 1950s ensemble. Other comments focused on the inferred division between Mother Nature and the group of younger women being stalked, possibly due to jealously on the part of Mother Nature. Depicted as an outsider, the actor cast as Mother Nature was consistently on the periphery of the younger, “more popular” group [P8], that of “a nerdy woman” [P3] unable to break the social boundary of being accepted “by the smart girls who were cool” [P7].

Several of the participants questioned how this negative depiction of menstruation might influence much younger girls in the context of developing self-esteem. In response to an interview probe to expand this discourse a little more, one participant [P10] explained,

I see that as a very negative thing as little girls grow up … I am very concerned about their self esteem and confidence because they see these beautiful women in these birth control advertisements, as compared to how they make menstruation look as Mother Nature, and might associate that the pill with being really pretty … or having a good outcome in life.

In addition to self-esteem and general well-being, the association between emotions and menstruation was also felt to be constructed as negative in the commercials and possibly influential to younger girls who are entering the developmental phase where societal norms around female roles gain greater importance. This last message was felt to be conveyed by the commercials choice of actors, as highlighted by a quote from one of the participants [P7],

the logical, intelligent looking one, the one that is not emotional, might appeal to a lot of girls, because in today's society there are increased expectations to be mothers, sisters, good workers and increase our productivity in today's busy society. That is hard to do if you are too emotional.

The terms “too emotional” and “emotional” were prominent in the transcriptions linking this theme to the earlier one of what discourses surrounding knowledge holders. One participant [P3] explained it this way,

the logical one is wearing a very nerdy outfit and is also carrying the laptop and doing research on seasonique … whereas the emotional woman is more free spirited and is wearing a dress, she is also dancing and just looks like she wants to have a good time.

### Theme 3—Female failings, fat, and feelings

Multiple discourses surrounding female failings accompanying the decision of whether to embrace or shun menstruation emerged as a central theme within the interviews. Discourses for embracing menstrual suppression were linked to an overarching narrative of pleasing or conforming to the image established by one's male partner when it came to menstruation. While all 10 of the participants noted genderized discourses during their interviews, the shape in which these discourses assumed varied. One participant [P9] noted an unspoken yet inferred message within the commercials, such as, “men don't want to know certain things that might destroy his perfect image of you” identified as blood spots on clothing, specifically white clothing that metaphorically suggests purity and privilege. Aside from the keeping of clothing free of menstrual blood, another participant [P2] also noted that such discourses extended to certain behaviors, as in “woman should look a certain way and act a certain way,” with another participant [P1] relating this to language, as in what to avoid saying, “they [women] should not be disgusting and talk about blood or menstruating”. A meaning that was shared across the interviews was the message of concealment that extended beyond one's intimate partner, from [P 10] “hide your faults from your man” to [P8] “from men in general.” A probing question used by one of the interviewers to seek deeper meanings regarding the function of such discourses resulted in the following explanatory statements; “women not being good enough” [P2] and “You really have to look deep in the advertising … if you are a woman they make you feel you have to be a certain way, and if you don't, you fail” [P5]. The theme of menstrual suppression commercials reinforcing genderized insecurities was also related to that of weight.

The intersection of female failing through weight and menstruation was in the context of how failing to suppress menstruation can lead to bloating causing temporary weight gain. This is illustrated by a comment from [P6] who said, “it [the commercial] plays on controlling your weight and water retention … it feeds on how women are told that they should be thinner.” The commercials’ emphasis on appearing distended because of menstrual bloating was felt to be greatly exaggerated, promoting self-surveillance as a form of self-control as noted by one participant's [P4] statement, “they always focus on bloating, the bloating, a lot of bloating is really bad and you don't want to bloat so you for sure, you should control that.” Seven of the participants noted that while the emphasis on bloating belonged on the spectrum of personal failings, it was one that could and should be controlled through menstrual suppression. One participant [P1] made visible the association between bloating and weight in her statement, “they play on controlling your weight, like with water retention. That seems to feed on how women are told that they should be thinner, so the ad is saying that in an indirect way, buy our product.” Likewise, the link between inconvenience and menstruation was also felt to be out of proportion in the commercials by [P2], as in,

so you're having your period and you know you can stain clothes and you have to buy pads or tampons or whatever, but it's a natural process. It's not ruining my life … like I can never wear white again!

There was a noted shift in perspectives after the participants watched the menstrual suppression commercials, from one of health and well-being to that of experiencing negative feelings. For example, the majority of participants expressed receiving an inferred feeling of guilt from the three commercials, meaning that by choosing to have 12 periods a year they were defiantly resisting the available option to be fully functional members of society. While no such statement was directly made by any of the actors in the commercials, there was consensus between the interviews that the choice to continue with monthly menstrual cycles was “wrong,” as illustrated by a statement made by [P3], “they say like, if you only got four periods per year then you could live your life to the fullest.” Another participant [P10] noted that by choosing to produce 12 periods a year rendered her “not productive” according to the commercials inferring that she was failing to be a contributing member of society. One participant [P1] summed it up by saying, “I got the impression that if you only got four periods a year then you could live life to the fullest because you're not in pain and you're not too emotional.”

This last participant's quote highlights a dimension emerging from the theme of female failings in regard to emotions, specifically the parameters to proportion, for example, what is too much as compared what to normal. Nine participants noted that the commercials framed “being emotional” as unnecessary and avoidable by taking either Yaz or Seasonale. One participant [P3] stated it this way that by taking menstrual suppression products, she could have “a miracle drug for suppressing both my period and my emotions.” The quest to find the transitory line between conforming to societal and cultural ideals of femininity which adheres to some emotion, while not being viewed as too emotional lest being labeled as irrational, was captured by one of the participants [P6] who said,

the ads are tapping into that insecurity of how women can become too emotional and how they can be irrational when they are emotional, so I'm not sure how we are supposed to figure this out, or if we should even try.

### Theme 4—fear or freedom?

The fourth theme that emerged from the transcriptions was that of fear versus freedom as a result of using menstrual suppression products. While the message of “menstrual freedom” was readily heard by the participants, 8 of the 10 women rejected this message for how it was so closely aligned to the DSM 5 diagnoses of PMS and PMDD, which created a climate of fear. The promise of freedom was conversely interpreted by these women as somewhat veiled and exploitative, which in turn resulted in a feeling of mistrust. Several of the participants mentioned that the linking of the terms menstruation, menstruation suppression, PMS, and PMDD may generate confusion for viewers as explained by [P5];

the question is whether or not you see a clear distinction between the definitions of PMS and PMDD—I'm not sure what they are saying. Is PMS a more severe version of PMDD? And is that because you are having periods?

It was also mentioned that this ensuing confusion may result in some women making decisions that were uniformed because of fear.

The two participants who did not outright reject this message expressed feeling uncertain as to the risks versus the benefits of menstrual suppression. For example, one participant [P2] felt that not having regular periods may be an advantage when playing competitive sports although she expressed concerns over the long-term absence of estrogen, “I have osteoporosis in my family, so, I would be worried about that later on.” The cost factor of menstrual suppression did appeal to the other participant [P10] who wondered as a student if it would be less costly having fewer periods.

Weighing the medical risks associated with taking menstrual suppression products as compared to possibly experiencing PMS or PMDD symptomatology caused both tension and confusion on the part of the majority of the participants. All 10 participants felt that the commercials created a binary of options, with no grey in which to navigate such an important decision. One of the participants [P8] summed this last theme up with the statement “women watching these commercials may go through periods of self-doubt … they may think they have this psychiatric disorder but they don't … but what if they do and feel they need to take these pills?”

## Discussion

The purpose of this study was to explore young adult women's experiences and understanding of menstrual suppression through the viewing of menstrual suppression advertisements. The overarching questions we explored in this paper were: What are the meanings attached to menstrual suppression by young women? How do these meanings inform their decision-making? We define menstrual suppression in this article as the use of oral contraceptives to prevent menstruation from occurring either in its normal frequency or interrupting the process for extended periods of time.

While this topic is not new, several of the findings from our study offer different or more current understandings from earlier research findings.

A study with a similar participant group but conducted in 2003 titled *To Bleed or not to Bleed: Young Women's Attitudes toward Menstrual Suppression* identified as a key finding women who perceived menstruation as bothersome were more supportive of suppression (Johnston-Robledo et al., 2003). Despite experiencing menstrual symptoms of bloating and cramps, the majority of our participants stated that they were suspicious of the menstrual suppression commercials and would not consider medically suppressing menstruation that they viewed as “natural” and “healthy.” Many of the perspectives captured by the interviews can be captured by the statement by participant [P3];

I don't really see the point [of menstrual suppression]. Yeah, having your period is inconvenient, but, it isn't that big of deal. Taking artificial medication or hormone is. I know my body is healthy when it bleeds every month and I would be very concerned if that didn't happen.

Similarly, unlike the previous study, the majority of participants felt that the “messiness” or unpleasant menstrual symptoms were exaggerated in the commercials and did not reflect their personal experience with menstruation. One participant [P2] was unsure, citing the reason to consider using menstrual suppression products was toward an “increased quality of life without any pain or mess.”

Another finding from our study extends the literature in how the use of metaphors is used regarding menstrual suppression marketing by pharmaceutical companies. Chrisler (2011) discussed how “bellicose metaphors about the body” positions women to be at war with their menstrual cycle, in battle with hormones or perpetually engaged in a conflictual state with those traits that characterize one as female, such as emotions (p. 202). In this vein, the combat is situated between the woman and her bodily processes, suggesting that menstrual suppression will free her of menstrual captivity. Participants in our study viewed the metaphor of division and conflict differently, not against their body but instead against each other. There was a consensus that the commercials skillfully targeted the gendered characteristic of insecurity to a possible one of belonging and acceptance. Tensions played out in the commercials between the “cool group” and “the outsiders” heightened the primal human fear of being left out or ostracized. One of the participants [P1] captured this beautifully when she stated “they are promoting one type that everyone feels they need to fit into … that's a really unfair portrayal of women.” When asked to elaborate a little more on possible reasons why menstrual suppression commercials might aim for this oversimplication, she stated “people, like women, are really complicated, but the commercials want to make using pills really simple, like “don't think too hard, and don't ask too many questions.”

An extension of the battle theme is to explore who belongs to what camp and how the commercials actually replicate infancy tactics of retreat, camouflage, and formation. In the Seasonale commercial, menstruation is personified by an older woman wearing the color of camouflage, green, who ritually engages in surprise offensive attacks on small groups of women who then collectively retreat seeking refuge. Another variation of the battle camps metaphor sees the categorization of bubbly, high-pitched, blonde-haired women positioned against serious, slow-speaking, brunette-haired women with knowledge heavily weighted in favor of the latter group. As knowledge holders, this group of women was more likely to be cast in the role of physician and lawyer, perceived to be more credible and trustworthy than their blonde counterparts (Takeda, Helms, & Romanova, 2006). The only shared commonality detected between these two groups by the participants was that of being “fair skinned,” another inference regarding race and who can be trusted (Nunnally, 2012).

This absence of colored women as knowledge holders is what Mahdawi (2015) refers to as the “whitewashing” in advertising. A 2014 study exploring diversity in advertising by the University of Toronto found that 87% of actors were white, with commercials reproducing “narrow culture schemas” that reinforced dominant associations between color, socio-economic class, and education (Baumann & Ho, 2014, p. 164). The absence of racial diversity in the menstrual suppression advertisements reflects Baumann and Ho's “white natural” and “white highbrow” cultural schemas (pp. 161–162) inferring that only white, educated, women of a higher socio-economic class are interested in menstrual hygienic practices. This proposition is best exemplified in the Seasonale commercial where all the actors are white, young women frolicking amidst a lush, tropical holiday setting masking the underlying linkages of race, age, and monetary power. The scene projects an image of success, a value revered in Western society. However, it also underscores a dangerous inverse relationship; choosing to remove menses leads to a higher probability of success.

Lastly, our study extends and augments theoretical suppositions offered by Casper and Moore (2009) and Gailey (2014) regarding the body, but within the specific context of menstrual suppression: these include the privileged body and the menstrual body where female and fat intersect.

In her book, *The Hyper (in)visible Fat Women: Weight and Gender Discourse in Contemporary Society*, Gailey (2014) includes a section on the privileged body. Privileged bodies are those that are invisible as compared to “visually obstructive bodies” who become visible “at inopportune times” (p. 8). While Gailey's definition stays within the parameters of socially acceptable gendered behaviors such as not talking too loud or taking up too much physical space, we also see her definition extending to menstruation. Privileged bodies are also those that do not leak socially offensive liquids such as blood. A central message within the menstrual suppression commercials is the “avoidance of embarrassing leaks” and “the freedom to do whatever you want.” While other feminists have made the link between the signs of menstruation and societal taboo (Docherty, 2010; Grosz, 1994), the majority of participants in our study refuted the claim that blood spotting as a visible result of menstruation is shameful. As one participant [P7] stated, “blood is a sign of you are healthy and that you are able to reproduce. So, um, your period is a sign of health.” Another participant [P5] associated monthly bleeding to health, as in “it is a sign of health because you're regular and you're getting your period every month.” In this regard, our participants suggested that the privileged body is one that regularly makes visible the signs of menstruation including blood as it represents a sign of health. The connection between menstruation and health has also been noted by other researchers (Klaeson, & Bertero, 2008).

This last comment also links to Casper and Moore's (2009) discourse on surveillance or the act of observing others bodies for social cues about their position within the social hierarchy. Bodies leaking menstrual blood may speak to the lack of financial ability to purchase menstrual suppression aids, or a lower social and economic position. Several of our participants linked the statement “live life to the fullest” found in the Yaz and Seasonique commercials, to the educational achievements held by the females who spoke to the benefits of menstrual suppression. Hence, what was not said, but insinuated in the commercials, was the association between personal and professional success and the absence of a monthly cycle. Also insinuated was the association between menstruation and the appearance of “fat” due to menstrual bloating. While some temporal distention is normal during the follicular stage of menstruation, it is short-lived and does not warrant medical intervention. However, the mentioned commercials suggest that bloating can and should be controlled and the decision not to might be perceived as a failing on the part of the woman should she not strive to “always look your best” in addition to fully a contributing member of society.

One of the key findings that we spend time pondering was the universal degree of agency displayed by our participants in refuting the embedded messages of sexism, ageism, racism, classism, and gendered capitalism found in the commercials. We wondered whether the younger age of the participants (21.8 years) or the fact that they were all university students might be contributing reasons for their ability to decipher underlying messages of nuanced consumer coercion. Zeisler (2016) offered another explanation that speaks to the emergence of a feminist informed group, which finds the creation of “marketplace feminism” an affront to their personal values. She posits that the packaging of “empowerment” through capitalism suggests that the choice of whether to use body surveillance products should be enough, without first asking why such surveillance is even needed. We do not know if any of our participants subscribe to Zeisler's proposition, although their responses suggest agreement.

Lastly, we offer the reader a reflective view of how our two standpoints as mother and daughter informed our thinking, discussion, and writing on this topic. As a daughter I was able to easily connect and establish rapport with the women that we interviewed who were similar in age. Those who were using birth control pills as a lifestyle drug shared common insights and perceptions to my own, specifically the shaming of women as this reflected my own experiences. Doing this study as a mother and daughter gave me spaces to go beyond our biological relationship and delve into a topic that might have been either too difficult or uncomfortable during my mother's early adult years. I felt the process of writing this article provided multiple places for us to better understand something that we shared, yet from totally different temporal and knowledge locations.

As a mother, I attempted to be mindful of how my position as an older woman employed at a university might unduly influence the interviews with participants who were 30 years younger than myself, and if the topic may cause them some degree of hesitation in speaking with me. Being in the same age range as their mothers also made me wonder how this might inform their sharing of experience. In retrospect, it was the socially normative and negative family of origin messages received during my adolescence regarding menstruation that were juxtapositioned beside participant messages of menstrual embracement and agency. I was impressed at how these women embraced their lived experiences of menstruation with clarity and conviction.

This article also created space to compare and contrast our individual experiences on how menstruation was presented and marketed to us as women, reflecting the shifting discourses on femininity and how our experiences related to those of the participants. For example, as a daughter the topic of menstruation was openly discussed as a monthly normality in our household that consisted of three other females. This compared to my mother's experience where menstruation was tidily packaged in a pink Kotex box secretly hidden behind cleaning products in a bathroom cabinet. As a mother, monthly periods were best not spoken about; as such topics were insinuated to be either “dirty” or better contained to the (male) doctor's office. This contrasted to observing the comfort level expressed by my daughter and her friends when talking about their periods or not leaving the room when a commercial on menstrual hygiene products come on television. As a daughter, the absence of shame and embarrassment associated with menstruation separated my lived experience from my mother's. These opposing narratives undoubtedly shaped our reflexivity during the interviews, from a comfort and attunement level with our participants to data analysis, where the discourses around certain words embodied diverse meanings based upon our personal histories.

## Conclusion

This research suggests that menstrual suppression commercials reinforce gendered stereotypes of women and greatly exaggerate the negative symptoms associated with menstruation based upon the lived experiences of our participants. With the exception of one participant who would take menstrual suppression products for reasons of convenience and comfort, the majority or other nine women found the messages embedded in the three commercials to be dismissive and divisive to women in general. The insinuated negative constructions around the words “healthy” and “lifestyle” conflicted with the values held by the participants and raised suspicions as to the safety of the product. We feel this study raises even more questions around current constructions of ageism, racism, gender, and capitalism and how such constructions intersect in the context of menstrual suppression. We encourage reflexivity on the part of the reader regarding personal schemas of menstruation, and how such narratives have informed current practices of menstruation. The temporal landscape of menstruation has changed within medical, social, cultural, and legal contexts due to pockets of resistance by women who refuse to be commodified. Identifying and exploring small sites of agency is a critical process toward collectively challenging patriarchal constructions of menstruation as unnatural, unhealthy, or simply unwanted.
